# Assessing the effect of starch digestion characteristics on ileal brake activation in broiler chickens

**DOI:** 10.1371/journal.pone.0228647

**Published:** 2020-02-07

**Authors:** Eugenia Herwig, Karen Schwean-Lardner, Andrew Van Kessel, Rachel K. Savary, Henry L. Classen

**Affiliations:** Department of Animal and Poultry Science, University of Saskatchewan, Saskatoon, Saskatchewan, Canada; University of New England, AUSTRALIA

## Abstract

The objective of this research was to evaluate activation of the ileal brake in broiler chickens using diets containing semi-purified wheat (WS; rapidly and highly digested) and pea (PS; slowly and poorly digested) starch. Diets were formulated to contain six WS:PS ratios (100:0, 80:20, 60:40, 40:60, 20:80, 0:100) and each starch ratio was fed to 236 Ross 308 male broilers housed in 4 litter floor pens. At 28 d of age, the effect of PS concentration was assessed on starch digestion, digestive tract morphology, and digesta pH and short-chain fatty acid (SCFA) concentration. Glucagon-like peptide-1 (GLP-1) and peptide tyrosine-tyrosine (PYY) status were assessed in serum (ELISA) and via gene expression in jejunal and ileal tissue (proglucagon for GLP-1). Data were analyzed using regression analyses, and significance was accepted at *P* ≤ 0.05. Increasing dietary PS resulted in reduced starch digestibility in the small intestine, but had no effect in the colon. Crop content pH responded quadratically to PS level with an estimated minimum at 55% PS. Total SCFA increased linearly in the crop with PS level, but changed in a quadratic fashion in the ileum (estimated maximum at 62% PS). Ceacal SCFA concentrations were highest for the 80 and 100% PS levels. The relative empty weight (crop, small intestine, colon), length (small intestine) and content (crop jejunum, Ileum) of digestive tract sections increased linearly with increasing PS concentration. Dietary treatment did not affect serum GLP-1 or PYY or small intestine transcript abundance. In conclusion, feeding PS increased the presence of L-cell activators (starch, SCFA) and increased trophic development and content of the digestive tract, suggestive of L-cell activation. However, no direct evidence of ileal brake activation was found by measuring venous blood levels of GLP-1 or PYY or corresponding gene expression in small intestine tissue.

## Introduction

In its simplest definition, starch is a polymer of glucose molecules linked by 1–4 and 1–6 α-glycosidic bonds [1]. However, its physicochemical characteristics such as the size of granules, degree of crystallinity, amylose-amylopectin ratio and the presence of other compounds can affect its rate and extent of digestion [2]. These differences in digestibility have resulted in a classification method for starch, which is based on its rate of *in vitro* digestibility. Rapidly digested (RDS), slowly digested (SDS) and resistant (RS) starch classifications are based on digestion times of under 20 min, 20–240 min and over 4 h, respectively, using the *in vitro* procedure described by Englyst et al. [3]. Although developed to mimic starch digestion in mammals, the general concept of starch digestion categories also applies to other monogastric animals, including chickens.

Traditional starch sources included in poultry diets (e.g. wheat, corn) are rapidly digested and also highly digestible. However, a number of studies have shown that the incorporation of starch with a lower rate and extent of digestion in poultry diets can be beneficial, particularly in terms of feed efficiency [4–8]. Little is known regarding the reasons for the observed effect. In mammals, undigested nutrients in the distal small intestine, particularly fats and carbohydrates, result in the activation of enteroendocrine L-cells (L-cells), which stimulate the release of glucagon-like peptide 1 [GLP-1; [9–11]9–11] and peptide tyrosine-tyrosine [PYY; 12,13]. These peptides are proposed to reduce gastric emptying, which in turn increases digestibility and inhibits feed intake by enhancing satiety [14,15], activating a mechanism known as the ileal brake. Glucagon-like peptide-2 (GLP-2), it is also released from L-cells along GLP-1 and PYY [16,17]. The function of GLP-2 has been linked to mucosal hyperplasia [18–20]. However, conclusive proof of these mechanisms acting in chickens, particularly the effect on satiety, is lacking.

In mammals, L-cells are commonly found in the distal small intestine and the colon [21]. However, in chickens these cells are located mainly in the jejunum and ileum, with just a few in the duodenum and none in other sections of the digestive tract [22,23]. Little research has been conducted on the type of nutrients that activate GLP-1 or PYY release in chickens. Monir et al. [24] found a direct relationship between the frequency of occurrence of GLP-1 containing cells and dietary protein concentration. In another study, the effect of feed intake on GLP-1 immunoreactive cells in broilers was assessed [24]. Contrary to what has been found in mammals, the occurance of GLP-1 immunoreactive cells in chickens increased under low feed intake. On the other hand, PYY acts in a manner that resembles the one reported in mammals, with increased gene expression under *ad libitum* feeding when compared to fasting [23]. Both GLP-1 and PYY appear to affect feed intake in chickens as well. Feed intake of day-old broilers and laying hen chicks was reduced when they were administered with a central injection of GLP-1 [25,26]. A peripheral injection of PYY in 9 day-old broilers had a similar effect [27]. Thus, many of the mammalian ileal brake components have been confirmed in chickens, but some differences warrant precaution when drawing conclusions.

To understand the impact of starch digestion characteristics on digestive tract morphology and physiology, diets used in the experiments described in this article were formulated to contain variable proportions of semi-purified wheat (WS; rapidly digested) and pea (PS; slowly digested) starch and fed to broiler chickens from 0 to 28 d of age. More specifically, the research objective was to evaluate the effect of starch digestion on activation of the ileal brake. It was hypothesized that the presence of starch along the digestive tract would increase with concentration of dietary PS. In addition, starch digestion and fermentation products would be sensed by L-cells, resulting in activation of the ileal brake mechanism. The presence of starch and carbohydrate fermentation (as reflected by pH and short-chain fatty acid (SCFA) concentration) were measured to assess L-cell activation potential, while digestive tract morphology, digesta content, feed passage rate, serum concentrations of GLP-1 and PYY and small intestine abundance of proglucagon (GLP-1 precursor) and PYY transcripts were measured to determine if activation had occurred.

## Materials and methods

This work was approved by the University of Saskatchewan’s Animal Research Ethics Board and adhered to the Canadian Council on Animal Care guidelines for humane animal use [28,29].

### Experimental treatments

To study the effect of starch digestion on the activation of the ileal brake, six diets were formulated to be identical (Table 1), with the exception of the source of the starch fraction, which contained graded levels of semi-purified WS and PS ranging from 100% WS to 100% PS. Semi-purified WS (Archer Daniels Midland Company, Montreal, QC, Canada) and PS (Parrheim Foods, Saskatoon, SK, Canada) were chosen after confirming differences in starch digestion rate between sources via *in vitro* analysis [7]. In accordance with previous research using intact wheat and pea [30], PS was found to be digested more slowly and to a lesser extent than WS. Because the purity of the starch sources was not equal (WS = 92.5% *vs*. PS = 80.0% starch), purified pea protein (Parrheim Foods, Saskatoon, SK, Canada) or a combination of purified pea protein and purified pea fibre (Parrheim Foods, Saskatoon, SK, Canada) was added to the WS to equalize starch, protein and fibre concentrations. In Experiment 1 (floor trial), the starch fraction of the 0% PS diets contained 87% WS and 13% pea protein, while in Experiment 2 (cage trial), the starch fraction of the same diet contained 80% WS, 11% pea fibre and 9% purified pea protein. Thereafter, in both experiments, the appropriate amount of this mixture (20, 40, 60, 80 or 100%) was replaced by PS to produce the rest of the treatments. Diets were formulated to meet or exceed the Aviagen nutrient requirements for broiler chickens [31]. The feed was manufactured at the Canadian Feed Research Centre (North Battleford, SK, Canada). Diets were pelleted with conditioning temperatures not exceeding 85°C; starter diets were crumbled prior to feeding. An indigestible marker (0.03% TiO_2_) was added on top of the formulation from d 24–28 to permit measurement of starch digestibility (Finisher diet. Experiment 1) and digesta passage rate (Experiment 2).

**Table 1:** Ingredient composition and nutrient content of treatment diets.

	Experiment 1			Experiment 2
Ingredient (%)	Starter	Grower	Finisher	
Semi-purified starch	47.49	53.66	59.35	58.19
Soybean meal	39.08	32.65	27.31	27.51
Porcine meal	5.00	5.00	5.00	5.00
Oat hulls	2.00	2.00	2.00	2.00
Soybean oil	2.71	3.50	3.36	3.07
Monocalcium phosphate	0.97	0.78	0.68	0.75
Limestone	1.19	0.92	0.86	0.88
Sodium chloride	0.37	0.37	0.37	0.37
Vitamin/mineral premix^1^	0.50	0.50	0.50	0.50
Ameri-Bond 2x^2^	0.00	0.00	0.00	0.50
Choline Chloride	0.10	0.10	0.10	0.10
DL-Methionine	0.52	0.46	0.42	0.49
L-Threonine	0.07	0.06	0.05	0.18
L-Isoleucine	0.00	0.00	0.00	0.05
L-Valine	0.00	0.00	0.00	0.13
**Calculated nutrient composition (%)**				
AME (kcal/kg)	3,025	3,150	3,200	3,100
Dry matter	88.61	87.99	88.22	88.79
Crude protein	25.09	22.43	20.24	19.73
Crude fat	4.34	5.14	5.01	4.05
Calcium	1.05	0.90	0.85	0.87
Available phosphorus	0.50	0.45	0.42	0.44

AME: apparent metabolizable energy.

Starter (0.4 kg/bird) and grower (1.4 kg per birds) were fed based on chick placement; the finisher diet was subsequently fed until the end of the trial.

^1^Supplied per kilogram of diet: vitamin A (retinyl acetate + retinyl palmitate), 11000 IU; vitamin D_3_, 2200 IU; vitamin E (dl-α-tocopheryl acetate), 300 IU; menadione, 2.0 mg; thiamine, 1.5 mg; riboflavin, 6.0 mg; niacin, 60 mg; pyridoxine, 4 mg; vitamin B_12_, 0.02 mg; pantothenic acid, 10.0 mg; folic acid, 0.6 mg; biotin, 0.15 mg; copper, 10 mg; iron, 80 mg; manganese, 80 mg; iodine, 0.8 mg; zinc, 80 mg; selenium, 0.3 mg; calcium carbonate, 500 mg; antioxidant, 0.625 mg; wheat middlings, 3772.73 mg.

^2^Lignin sulfonate dehydrated (LIGNOTECH Florida LLC, Fernadina Beach, FL).

### Birds and bird housing

#### Experiment 1

A total of 1,416 Ross x Ross 308 male broiler chicks were obtained from a commercial hatchery (Sofina Inc., Wynyard, SK, Canada) and housed in two independent rooms at the University of Saskatchewan Poultry Centre (Saskatoon, SK, Canada). Chicks were allocated randomly to six dietary treatments with two replications per room.

Birds were raised under environmentally controlled conditions of light (23L:1D, 18 lux for 2 d followed by 16L:8D at 10 lux until the end of the experiment) and temperature (starting at 33°C) was reduced daily to reach 21°C by d 25. Light was provided via incandescent light bulbs and each room was heated with hot water pipes running along three walls. Feed and water were offered *ad libitum*. Wheat straw was used as bedding material in floor pens (4.6 m^2^). Each pen was furnished with a tube feeder (36 cm diameter from 0–21 d and 43 cm diameter thereafter) and one nipple drinker with six nipples. A supplemental feeder and drinker were provided during the first two days of the experiment. Groups of 59 chicks were allocated to each pen.

#### Experiment 2

One hundred eighty newly hatched Ross x Ross 308 male broiler chicks were obtained from a commercial hatchery (Sofina Inc., Wynyard, SK, Canada). Chicks were housed randomly in groups of six into 36 conventional cages (433.5 cm^2^ per bird, 51 cm feeder trough, two adjustable 360° nipple drinkers) at the University of Saskatchewan Poultry Centre (Saskatoon, SK, Canada), resulting in six replications per dietary treatment.

During the first three days, the photoperiod was set at 23L:1D, followed by a 18L:6D photoperiod until the end of the experiment. The photophase light intensity was 30–40 lux during the entire trial. Light was provided using incandescent light bulbs and included dawn and dusk periods of 15 min each. Heat was provided via hot water pipes running along three walls of the room. Temperature was set to start at 32°C and gradually cool down to reach 21°C by 25 d. Feed and water were offered *ad libitum*, and they were provided via chick feeder and feed trough, and ice cube trays and nipple drinkers, respectively, for 3 d, after which the chick feeder and the ice cube tray were removed and only cage feed trough and nipple drinkers remained.

### Data collection

#### Experiment 1

At 28 d of age, four h after lights came on, seven random birds per pen were individually weighed and euthanized via intravenous injection of T61 Euthanasia solution (0.35ml/kg body weight; Merck & Co., Inc. Kirkland, QC). The digestive tracts were removed from four birds per replication. *In situ* pH was assessed in the crop, ileum and caecal contents using a pH Meter (model Phi 34, Beckman Instruments, Inc.; Fullerton, CA, USA). Each digestive tract was sectioned into crop, proventriculus, gizzard, duodenum, jejunum, ileum, ceca and colon. The jejunum and ileum were further divided into proximal and distal sections. Full and empty weights of each section were recorded, along with the length of small intestine, caeca and colon sections. Digesta content weight was determined by subtracting the empty weight from the full tissue weight of each section. Weights were also obtained for the heart, liver and pancreas. All digesta content per section, except from the proventriculus and gizzard, were collected, pooled per pen and frozen immediately for starch and titanium determination.

Prior to euthanasia, peripheral blood (brachial vein) was collected from the three remaining birds, for determination of serum concentrations of GLP-1 and PYY using ELISA. Samples were allowed to clot and centrifuged for 15 min at 1000 x g. Resulting sera were stored (-20°C) until further analyses. Following euthanasia and digestive tract removal, digesta content from the crop, distal ileum and caeca were collected into 15 mL centrifuge tubes, sealed immediately and frozen (-20°C) for SCFA analysis. Mid-jejunum and mid-ileum sections (1 cm long) were also collected, immediately frozen in individual bags using liquid nitrogen, and stored at -80°C for PYY and proglucagon gene expression analyses.

#### Experiment 2

At 28 d of age, feed passage rate was determined following a modified version of the procedure described by Salih et al. [32]. After removing feed the previous night from 6 pm to 4 am (10 h), birds were fed with a known amount of test diets supplemented with 0.3% TiO_2_ for 2 h. At the end of this period, birds were immediately returned to the unlabeled test diets, and the remaining feed per cage was weighed to calculate titanium consumption on a replication basis. Total excreta per cage was collected into individual bags every 30 min, from the moment TiO_2_ supplemented diets were provided, by placing clean aluminum trays under each cage. Excreta collection continued for 13 h and samples were individually weighed and frozen for future titanium determination.

### Lab analyses and calculations

#### Dietary analysis

Diets were analyzed for starch (TS), moisture, fat, crude protein (CP), ash, dietary fibre (DF) and titanium following AOAC standard methods [33]. Starch was determined using the Megazyme Total Starch Kit (Megazyme Inc., Chicago, IL, USA) following method 996.11. Moisture was measured following method No. 930.15. Fat content was determined by ethyl ether extraction using Goldfish Extraction Apparatus (Labconco model 35001; Labconco, Kansas, MO, USA) following method 920.39. Nitrogen was determined using a Leco N analyzer (Leco FP-528; Leco Corp., St. Joseph, MI, USA; method No 990.03) and the value multiplied by 6.25 to obtain CP. Ash was measured (method 942.05) using a muffle oven (Lindberg/Blue BF51842, Asheville, NC 28804, USA). Dietary fibre was calculated by the addition of the soluble and insoluble fractions, which were determined using a Megazyme Total Dietary Fiber kit (Megazyme Inc., Chicago, IL) following method 985.29. Titanium dioxide was determined using the procedure described by Myers et al. [34]. All dietary measurements were assessed in duplicate except for starch which was measured in triplicate.

#### Starch digestibility

*In vivo* starch digestibility was calculated by measuring starch, titanium and dry matter in the feed, proximal and distal jejunum and ileum, and colon digesta contents. Starch, moisture and titanium were determined as previously described for feed analysis.

#### Short-chain fatty acid analysis

Determination of SCFA was performed following a modified version of the procedure described by Zhao et al. [35] measuring the samples in triplicate. Briefly, an internal standard was prepared by diluting 0.5 g of 3-methyl-n-valeric acid in 1 L of 0.15 mol/L of oxalic acid. The standard solution was made by weighing 400 mM of acetic, propionic and butyric acid, 200 mM of isovaleric, valeric and lactic acid, 50 mM of caproic acid and 100 nM of isobutyric acid. After thawing, samples of digesta were weighed in triplicate and then 25% phosphoric acid was added 1:1, mixed and centrifuged (12,500 x g for 5 min). One mL of supernatant in triplicate was collected and placed into microcentrifuge tubes. The internal standard was added at 1:1 ratio to supernatant and centrifuged (12,500 x g for 10 min). Samples were filtered using a 3 mL syringe and a 0.45 μm Nylon filter into a glass GC vial. Concentrations of SCFA were measured using a Thermo Scientific Gas Chromatograph (model Trace 1310, Milan, Italy) and a Zebron capillary GC column (ZB-FFAP, length: 30 m, I.D: 0.25 mm, film thickness: 0.25 μm, Phenomenex, Torrance, CA).

#### Determination of GLP-1 and PYY serum concentrations

The concentration of GLP-1 in serum was measured using Chicken GLP-1 ELISA kit (Catalogue No: E-EL-Ch0160; Elabscience Biotechnology, Wuhan, China). PYY serum concentrations were determined using Chicken Peptide YY ELISA kit (Catalog No: CSB-EL019128CH, Cusabio Biotechnology, Wuhan, China).

#### Gene expression

Tissue samples collected from jejunum and ileum were first individually homogenized using mortar and pestle while still frozen under liquid nitrogen. RNA extraction of the samples was performed using TRIzol reagent Ambion (Invitrogen, Carlsbad, CA) and cDNA was prepared using a High-Capacity Reverse Transcription kit (Applied Biosystems; Thermofisher Scientific, Foster City, CA). The reaction was performed at 25°C for 10 min, after which the temperature was increased to 37°C for 2 h, and followed by 5 min at 85°C using a C1000 Touch Thermal Cycler (Bio-Rad Laboratories, Hercules, CA). The obtained cDNA was stored at -20°C.

Gene expression was measured using the quantitative Polymerase Chain Reaction (qPCR) method. A master mix was produced by using 10 μL of SsoFast Evagreen Supermix (Bio-Rad Laboratories, Hercules, CA), 0.8 μL of forward primer (10 μM) and 0.8 μL of reverse primer (10 μM) specific for chicken proglucagon, proglucagon B, PYY, GAPDH or RPS7 (Table 2; Invitrogen, Carlsbad, CA), and 6.4 μL of Nuclease-free water per well. Two μL of cDNA and 18 μL of master mix were added to qPCR plates in duplicate. A no-reverse transcriptase control and a no-template control were included in duplicate in each plate, as well as a standard curve, which was run in triplicate. Standard curves were assembled by using a concentration of 10 ng of cDNA per microliter for the first standard, followed by 5-fold dilutions. Each plate was set in the CFX Connect Optics Module Real-Time System (Bio-Rad Laboratories, Hercules, CA). Cycling conditions for all genes were identical including denaturation at 95.0°C for 5 min, and annealing and extension as 60.0°C for 5 min. Amplification efficiency for each gene ranged from 86 to 137% and R^2^ values for standard curves ranged from 94.5 to 99.7. Obtained values of proglucagon, proglucagon B and PYY were normalized by the average of GAPDH and RPS7, previous to conducting statistical analyses. Expression of reference genes was not affected by treatment.

**Table 2:** Primers used for quantitative polymerase chain reaction.

Protein	Sequence	Reference
Proglucagon	F: 5’-CACAAGGCACATTCACCAGT-3’	[36]
	R: 5’-TTCTTTGGCAGCTTGACCTT-3’	
Proglucagon B	F: 5’-CACAAGGCACATTCACCAGT-3’	[36]
	R: 5’-TGGTATTCTCCCAAAAGGTCTC-3’	
PYY	F: 5’-AGGAGATCGCGCAGTACTTCT-3’	[27]
	R: 5’-TGCTGCGCTTCCCATACC-3’	
GAPDH	F: 5’-GTGAAAGTCGGAGTCAACGGA-3’	[37]
	R: 5’-AAGGGATCATTGATGGCCAC-3’	
RPS7	F: 5’-TAGGTGGTGGCAGGAAAGC-3’	[38]
	R: 5’-TTGGCTTGGGCAGAATCC-3’	

F: Forward; R: Reverse

#### Rate of feed passage determination

Excreta samples were dried at 55°C using a forced air oven, weighed and ground. Titanium concentration in excreta was determined following the procedure described by Myers et al. [34]. Results were expressed as a percentage of titanium intake over 2 h. The time required for 50% of the consumed titanium appearance in excreta (T50) for each experimental unit was calculated by finding the best fitting curve (quadratic; R^2^ ≥ 0.98).

### Statistical analyses

Experiment 1 was designed as a randomized complete block design. To determine the significance of the room effect an ANOVA was run. As room had no significant impact, it was removed from the analyses. Regression analyses (Proc reg, linear regression; Proc Rsreg, quadratic regression) were performed with SAS statistical software (Version 9.4. SAS Institute Inc., Cary, NC). Normality of the residuals and homogeneity of variance was checked in all data before statistical analyses. No transformation was required for digestibility data. Due to differences in body size, all digestive tract data were reported as relative values to live body weight. In addition, because an effect of diet was found on duodenum, jejunum, ileum and caeca lengths, digesta content in this section was relativized by both, tissue length and body size. Logarithm transformations were applied to the digestive tract, ELISA and SCFA data when required to normalize the residuals. Similarly, the square root of gene expression data was used for statistical analyses. All differences were considered at *P* ≤ 0.05 and trends were considered when 0.10 ≥ *P* > 0.05.

## Results

*In vivo* starch digestibility was high for all diets, with over 77% starch digestion in the proximal jejunum and achieving almost 100% digestion in the colon (Table 3). The percentage of starch digestion decreased linearly as dietary PS increased for jejunum and distal ileum sections. Proximal ileum starch digestibility showed a quadratic response with the lowest value at 100% PS. A trend (*P* = 0.073) for starch digestibility to decrease linearly with increasing PS was found in the colon.

**Table 3:** Effect of the proportion of dietary wheat and pea starch on the cumulative percentage of apparent digested starch in broilers at 28 d of age.

	Diets						SEM	Regression	R^2^	Equation
	0^1^	20	40	60	80	100				
Proximal jejunum	87.0	82.5	79.6	77.9	78.6	77.9	1.013	L = 0.002	0.35	*y* = -0.08*x* + 84.77
Distal jejunum	95.7	92.0	89.0	88.2	89.6	85.3	0.796	L<0.001	0.59	*y* = -0.09*x* + 94.28
Proximal ileum	99.1	98.0	96.2	96.6	96.7	95.4	0.264	Q = 0.037	0.76	*y* = 3_x_10^-4^*x*2–0.06*x* + 98.94
Distal ileum	99.1	98.5	98.3	97.5	97.8	96.8	0.160	L<0.001	0.81	*y* = -0.02*x* + 98.97
Colon	98.3	98.2	97.9	97.5	98.3	97.6	0.095	NS	-	-

^1^ Diets denominations indicate the percentage of pea starch in the starch fraction of the diet.

SEM: pooled standard error of the mean; L: linear regression P value; Q: quadratic regression; number of replications = 4 pens.

The pH of the crop contents responded quadratically to PS concentration, reaching an estimated minimum at the 55% PS concentration (Table 4). The pH of the ileal and caecal content showed trends (both *P* = 0.064) to change quadratically with PS concentration, attaining an estimated minimum at 61% PS for the ileum and an estimated maximum at 38% PS for the caeca. However, these effects were mostly driven by the higher distal ileum pH found in birds fed 0% PS and the lower caecal pH measured in birds fed 100% PS.

**Table 4:** Effect of the proportion of dietary wheat and pea starch on crop, ileum and caeca content pH of broilers at 28 d of age.

	Diets						SEM	Regression	R^2^	Equation
	0^1^	20	40	60	80	100				
Crop	5.19	4.89	4.92	4.84	5.00	5.01	0.045	Q = 0.042	0.05	*y* = 9_x_10^-5^*x*2–0.01*x* + 5.14
Ileum	6.85	6.15	6.47	6.13	6.26	6.34	0.082	NS	-	-
Caeca	6.50	6.59	6.48	6.53	6.59	6.26	0.036	NS	-	-

^1^ Diets denominations indicate the percentage of pea starch in the starch fraction of the diet.

Data was applied natural logarithm transformation; SEM: pooled standard error of the mean; P = *P* value; Q: quadratic regression; number of replications = 4 pens.

On average, the total SCFA concentration was highest in the caeca (170 μmol/g of digesta) followed by the ileum (81.54 μmol/g) and the crop (59.19 μmol/g; Tables 5 to 7). The amount of total, acetic, propionic and lactic acids in the crop increased in a linear fashion with increasing dietary PS, but on a proportional basis, only iso-butyric and valeric acids (linearly reduced) were affected by dietary PS (Table 5). In the distal ileum, absolute values for total SCFA responded quadratically with an estimated maximum at 58% PS, while propionic acid increased linearly with increasing PS (Table 6). Propionic acid showed a trend to respond in a quadratic fashion, reaching an estimated maximum at 68% PS (*P* = 0.098). When analysed as a proportion of total SCFA in the ileum, acetic acid showed a trend (*P* = 0.086) to change quadratically with a minimum at 61% PS, while butyrate showed a trend (*P* = 0.094) to increase linearly. No lactic acid was found in the ileum of birds fed 0% PS. Although not statistically significant (*P* = 0.116), both the amount and the percentage of lactic acid in the distal ileum increased from 20 to 60% PS and then declined at higher concentrations.

**Table 5:** Effect of the proportion of dietary wheat and pea starch on SCFA concentration (μmol/g of digesta) and composition (%) in the crop contents of broilers at 28 d of age.

	Diets						SEM	Regression	R^2^	Equation
	0^1^	20	40	60	80	100				
*SCFA concentration (*μ*mol/g)*										
Total	54.5	56.9	58.0	60.6	61.7	63.5	0.99	L = 0.008	0.41	*y* = 0.09*x* + 54.8
Acetic acid	22.2	23.2	23.8	25.0	25.5	26.3	0.41	L<0.001	0.52	*y* = 0.04*x* + 22.3
Propionic acid	7.6	9.0	8.5	8.9	8.9	9.6	0.21	L = 0.019	0.23	*y* = 0.01*x* + 8.0
Iso-butyric acid	1.4	1.4	1.4	1.4	1.3	1.3	0.03	NS	-	-
Butyric acid	-	-	-	-	-	-	-	-	-	-
Iso-valeric acid	-	-	-	-	-	-	-	-	-	-
Valeric acid	1.4	1.4	1.4	1.2	1.2	1.1	0.06	NS	-	-
Caproic acid	0.6	0.6	0.5	0.6	0.6	0.5	0.02	NS	-	-
Lactic acid	21.4	21.5	22.3	23.6	24.2	24.7	0.56	L = 0.019	0.23	*y* = 0.04*x* + 21.07
*SCFA mixture composition (%)*										
Acetic acid	40.8	40.8	41.1	43.9	41.4	41.5	0.51	NS	-	-
Propionic acid	13.9	15.8	14.8	15.6	14.4	15.2	0.37	NS	-	-
Iso-butyric acid	2.6	2.4	2.4	2.4	2.1	2.0	0.06	L<0.001	0.41	*y* = -0.005*x* + 2.6
Butyric acid	-	-	-	-	-	-	-	-	-	-
Iso-valeric acid	-	-	-	-	-	-	-	-	-	-
Valeric acid	2.5	2.4	2.5	2.1	2.0	1.7	0.11	L = 0.006	0.30	*y* = -0.008*x* + 2.6
Caproic acid	1.1	1.0	0.9	1.0	1.0	0.8	0.04	NS	-	-
Lactic acid	39.1	37.8	38.3	41.4	39.2	38.8	0.67	NS	-	-

^1^ Diets denominations indicate the percentage of pea starch in the starch fraction of the diet.

SEM: pooled standard error of the mean; L: linear regression P value; NS: not significant; number of replications = 12 male broilers.

**Table 6:** Effect of the proportion of dietary wheat and pea starch on SCFA concentration (μmol/g of digesta) and composition (%) in the ileal contents of broilers at 28 d of age.

	Diets						SEM	Regression	R^2^	Equation
	0^1^	20	40	60	80	100				
*SCFA concentration (*μ*mol/g)*										
Total	67.2	78.8	84.2	96.7	93.4	74.7	3.82	Q = 0.025	0.27	*y* = -0.008*x*^2^ + 1.0*x* + 64.7
Acetic acid	60.2	60.7	63.5	61.3	63.8	59.1	1.11	NS	-	-
Propionic acid	4.7	6.3	8.4	9.9	9.4	8.1	0.70	L = 0.050	0.17	*y* = 0.04*x* + 5.8
Iso-butyric acid	0.5	0.6	0.9	0.4	1.2	1.0	0.13	NS	-	-
Butyric acid	-	1.7	1.5	1.0	2.2	1.4	0.29	NS	-	-
Iso-valeric acid	0.5	0.8	1.0	0.6	1.1	0.7	0.12	NS	-	-
Valeric acid	0.4	0.5	0.6	0.4	1.0	0.9	0.14	NS	-	-
Caproic acid	1.1	0.8	1.1	1.0	1.1	0.8	0.10	NS	-	-
Lactic acid	-	7.4	7.3	22.1	13.6	2.6	3.45	NS	-	-
*SCFA mixture composition (%)*										
Acetic acid	90.0	78.6	77.3	67.0	72.6	79.2	3.10	NS	-	-
Propionic acid	6.6	7.8	10.2	10.9	10.4	10.7	0.87	NS	-	-
Iso-butyric acid	0.7	0.6	1.0	0.4	1.3	1.4	0.14	NS	-	-
Butyric acid	-	2.0	1.6	0.8	2.3	2.0	0.29	NS	-	-
Iso-valeric acid	0.7	1.0	1.2	0.7	1.2	0.9	0.14	NS	-	-
Valeric acid	0.5	0.6	0.6	0.3	1.0	1.1	0.15	NS	-	-
Caproic acid	1.6	1.1	1.3	1.0	1.2	1.1	0.12	NS	-	-
Lactic acid	-	8.4	6.8	19.0	10.1	3.7	2.87	NS	-	-

^1^ Diets denominations indicate the percentage of pea starch in the starch fraction of the diet.

SEM: pooled standard error of the mean; L: linear regression P value; Q: quadratic regression P value; NS: not significant; number of replications = 12 male broilers.

**Table 7:** Effect of the proportion of dietary wheat and pea starch on SCFA concentration (μmol/g of digesta) and composition (%) in the caecal contents of broilers at 28 d of age.

	Diets						SEM	Regression	R^2^	Equation
	0^1^	20	40	60	80	100				
*SCFA concentration (*μ*mol/g)*										
Total	156	159	162	166	177	203	3.7	Q = 0.002	0.77	*y* = 0.006*x*2–0.2*x* + 159
Acetic acid	94	94	97	98	105	126	2.6	Q<0.001	0.79	*y* = 0.001*x*2–0.3*x* + 96
Propionic acid	30.7	31.6	30.8	32.9	35.2	32.7	0.56	L = 0.043	0.17	*y* = 0.03*x* + 30.7
Iso-butyric acid	4.7	4.9	1.6	5.0	5.6	4.1	0.16	NS	-	-
Butyric acid	16.0	17.4	17.7	19.2	19.0	29.4	1.51	L = 0.014	0.25	*y* = 0.1*x* + 14.5
Iso-valeric acid	4.6	4.7	4.5	4.9	5.3	4.2	0.13	NS	-	-
Valeric acid	1.5	4.7	4.5	4.9	5.1	4.8	0.07	NS	-	-
Caproic acid	2.0	1.9	2.0	2.0	2.0	1.5	0.06	NS	-	-
Lactic acid	-	-	-	-	-	-	-	-	-	-
*SCFA mixture composition (%)*										
Acetic acid	60.1	59.1	60.2	58.6	59.3	62.2	0.39	Q = 0.034	0.25	*y* = 0.0008*x*2–0.07*x* + 60.3
Propionic acid	19.6	19.9	19.2	19.8	19.9	16.3	0.38	Q = 0.039	0.34	*y* = -0.0007*x*^2^ + 0.05*x* + 19.3
Iso-butyric acid	3.0	3.1	2.9	3.0	3.1	2.1	0.11	Q = 0.035	0.33	*y* = -0.0002*x*^2^ + 0.01*x* + 2.9
Butyric acid	10.3	11.0	11.0	11.6	10.7	14.2	0.58	NS	-	-
Iso-valeric acid	2.9	3.0	2.8	2.9	3.0	2.1	0.09	Q = 0.025	0.39	*y* = -0.0001*x*^2^ + 0.01*x* + 2.8
Valeric acid	2.9	2.9	2.8	2.9	2.9	2.4	0.05	Q = 0.008	0.48	*y* = -0.0001*x*^2^ + 0.007*x* + 2.8
Caproic acid	1.3	1.1	1.2	1.2	1.1	0.8	0.05	Q = 0.027	0.44	*y* = -8_x_10^-5^*x*^2^ + 0.005*x* + 1.2
Lactic acid	-	-	-	-	-	-	-	-	-	-

^1^ Diets denominations indicate the percentage of pea starch in the starch fraction of the diet.

SEM: pooled standard error of the mean; L: linear regression P value; Q: quadratic regression P value; NS: not significant; number of replications = 12 male broilers.

Dietary treatments also affected the amount of total, acetic and butyric acids found in the caeca (Table 7). Butyric acid increased linearly with PS, but total and acetic acid changed quadratically reaching a minimum at 18 and 25% PS, respectively. In both cases, however, the quadratic effect was likely the result of the sharp increase in total and acetic acid observed in the 100% PS treatment samples. The effects on SCFA production in the caeca were also accompanied by changes in the proportional composition of the SCFA mixture. All acids except butyric acid were affected quadratically by PS level, presenting all critical values between 29 and 41% PS. These quadratic effects were likely the result of the sudden increases or decreases in the relative concentrations of SCFA observed in the caeca in the 100% PS treatment.

The relative empty weights of the crop, proventriculus, jejunum, ileum, caeca and colon increased linearly with PS concentration, while no effect was found for the gizzard and duodenum (Table 8). The relative weight of digesta content for the crop, jejunum and ileum increased linearly with PS concentration as well, while no effect was found for other digestive tract sections. Increasing the concentration of PS resulted in a linear increase in the relative length of all sections of the small intestine and caeca but did not affect colon length (Table 8). Heart (0.57%), liver (3.38%) and pancreas (0.24%) relative weights were not affected by diet PS concentration.

**Table 8:** Effect of the proportion of dietary wheat and pea starch on digestive tract empty weights, contents and small intestine length of broilers at 28 d of age relative to body weight.

	Diets						SEM	Regression	R^2^	Equation
	0^1^	20	40	60	80	100				
*Empty tissue weight* (% of body weight)										
Crop	0.32	0.33	0.33	0.32	0.37	0.37	0.007	L = 0.007	0.07	*y* = 6_x_10^-4^*x* + 0.31
Proventriculus	0.36	0.37	0.39	0.37	0.39	0.41	0.007	L = 0.040	0.04	*y* = 4_x_10^-4^*x* + 0.36
Gizzard	1.04	1.02	1.15	1.06	1.05	1.07	0.016	NS	-	-
Duodenum	0.58	0.59	0.64	0.60	0.61	0.61	0.011	NS	-	-
Jejunum	1.28	1.34	1.35	1.32	1.42	1.42	0.021	L = 0.032	0.05	*y* = 0.001*x* + 1.29
Ileum	0.93	0.95	1.02	1.02	1.03	1.04	0.017	L = 0.017	0.06	*y* = 6_x_10^-4^*x* + 0.66
Caeca	0.32	0.32	0.36	0.34	0.37	0.36	0.006	L = 0.022	0.06	*y* = 4_x_10^-4^*x* + 0.32
Colon	0.086	0.087	0.089	0.090	0.095	0.102	0.0017	L = 0.002	0.09	*y* = 2_x_10^-4^*x* + 0.084
*Length* (cm/100 g of body weight)										
Duodenum	1.59	1.64	1.61	1.69	1.80	1.66	0.021	L = 0.038	0.05	*y* = 0.001*x* + 1.60
Jejunum	3.66	3.94	3.86	3.97	4.25	4.19	0.048	L<0.001	0.14	*y* = 0.005*x* + 3.72
Ileum	3.64	3.97	4.03	4.06	4.26	4.29	0.055	L<0.001	0.14	*y* = 0.006*x* + 3.74
Caeca	1.52	1.59	1.61	1.58	1.73	1.70	0.022	L = 0.004	0.08	*y* = 0.002*x* + 1.53
Colon	0.26	0.28	0.27	0.26	0.28	0.28	0.005	NS	-	**-**
*Content* (% of body weight)										
Crop	0.50	0.72	0.66	0.91	1.17	1.45	0.079	L<0.001	0.17	*y* = 0.009*x* + 0.44
Proventriculus	0.05	0.06	0.07	0.05	0.10	0.10	0.010	NS	-	**-**
Gizzard	0.7	0.5	0.8	0.7	0.6	0.7	0.04	NS	-	-
Duodenum^2^	0.08	0.08	0.07	0.07	0.09	0.08	0.002	NS	-	**-**
Jejunum^2^	0.13	0.14	0.14	0.16	0.20	0.18	0.004	L<0.001	0.27	*y* = 7_x_10^-4^*x* + 0.12
Ileum^2^	0.12	0.12	0.13	0.15	0.15	0.14	0.026	L = 0.006	0.08	*y* = 3_x_10^-4^*x* + 0.12
Caeca^2^	0.10	0.08	0.10	0.08	0.12	0.10	0.004	NS	-	**-**
Colon	0.05	0.07	0.06	0.05	0.06	0.06	0.003	NS	-	-

^1^ Diets denominations indicate the percentage of pea starch in the starch fraction of the diet.

^2^ Content weights were controlled by length and body weight

SEM: pooled standard error of the mean; L: linear regression P value; NS: not significant; number of replications = 4 pens.

The average serum concentrations of GLP-1 and PYY in 28 old broilers were 1,651.5±1,183.9 and 361.8±65.4 pg/ml, respectively. Diet concentration of PS did not affect GLP-1 or PYY serum concentrations (Table 9).

**Table 9:** Effect of the proportion of dietary wheat and pea starch on GLP-1 and PYY serum concentrations (pg/mL) of broilers at 28 d of age.

	Diets						SEM	Regression	R^2^	Equation
	0^1^	20	40	60	80	100				
GLP-1	1650	1478	1964	1995	1277	1545	141.5	NS	-	-
PYY	369	366	332	368	375	361	7.8	NS	-	-

^1^ Diets denominations indicate the percentage of pea starch in the starch fraction of the diet.

SEM: pooled standard error of the mean; NS: not significant; number of replications = 12 male broilers.

The relative mRNA levels of target genes were not affected by starch type, with the exception of the expression of proglucagon in the ileum, which decreased linearly as PS concentration increased (Table 10).

**Table 10:** Effect of the proportion of dietary wheat and pea starch on relative^1^ mRNA levels of proglucagon, proglucagon-B and peptide tyrosine-tyrosine in jejunum and ileum samples of broilers at 28 d of age.

	Diets						SEM	Regression	R^2^	Equation
	0^1^	20	40	60	80	100				
*Jejunum*										
PG	0.9	1.3	0.5	1.0	0.8	0.6	0.09	NS	-	-
PGB	0.9	1.3	0.4	0.9	0.6	0.3	0.13	NS	-	-
PYY	1.2	1.5	0.8	1.0	1.0	1.0	0.08	NS	-	-
*Ileum*										
PG	1.5	1.5	1.3	1.3	1.1	1.3	0.05	L = 0.047	0.08	*y* = -0.001*x* + 1.2
PGB	0.9	0.9	0.7	0.9	0.7	0.8	0.04	NS	-	-
PYY	0.9	0.7	0.8	0.7	0.7	0.7	0.07	NS	-	-

^1^ Obtained values of proglucagon, proglucagon B and PYY were normalized by the average of GAPDH and RPS7

^2^ Diets denominations indicate the percentage of pea starch in the starch fraction of the diet.

PG: proglucagon; PGB: proglucagon-B; PYY: peptide tyrosine-tyrosine; SEM: pooled standard error of the mean; L: linear regression P value; NS: not significant; number of replications = 12 male broilers.

On average, 7.91h were required for 50% of the consumed marker to be excreted at 28 d of age (Table 11). Diet affected passage rate in a quadratic fashion (*P* = 0.027), reaching an estimated maximum at 61% PS inclusion.

**Table 11:** Effect of the proportion of dietary wheat and pea starch on the time (min) required to excrete 50% of indigestible marker in broilers at 28 d of age.

	Diets						SEM	Regression	R^2^	Equation
	0^1^	20	40	60	80	100				
Time (min)	544	536	481	406	478	498	14.2	Q = 0.027	0.22	*y* = -75*x*2 – 33*x* + 454

^1^ Diets denominations indicate the percentage of pea starch in the starch fraction of the diet.

SEM: pooled standard error of the mean; NS: not significant; number of replications = 6 cages.

## Discussion

Performance criteria from a simultaneous trial run in identical conditions [7] show that broilers met or exceeded Aviagen performance criteria [31]. Although dietary treatments were formulated based on the analysis of starch samples, the starch concentration of the PS used for feed manufacturing was lower than expected (61 vs. 80%). As a result, a gradual reduction of the analyzed starch content occurred as PS level increased (Starter = 42.14 ± 5.88; Grower = 44.68 ± 3.55; Finisher = 45.18 ± 2.55%; S1 Appendix). Diets were formulated to keep fat and fibre concentrations similar among them to avoid the possibility of ileal brake activation due to components other than starch in the diets [15,39]. Analyzed fat levels were similar among diets (Starter = 4.57 ± 0.13; Grower = 4.97 ± 0.14; Finisher = 5.09 ± 0.24%; S1 Appendix), while TDF concentrations increased with higher concentrations of PS. However, of note, the more fermentable soluble dietary fiber fraction was relatively stable among diets (Starter = 2.78 ± 0.19; Grower = 2.61 ± 0.36; Finisher = 2.40 ± 0.17%). In summary, dietary treatments contained similar levels of both fat and soluble fiber. Thus, it is unlikely that any effects regarding L-cell activation can be attributed to dietary components other than starch in this trial.

### L-cell activation potential

In vivo starch digestibility values in all sections of the small intestine were high compared to digestibility values of intact starch sources in the same sections [30,40]. This was likely the result of the starch purification process, as it reduces or eliminates other grain-associated compounds that could lower digestibility [41], and it can also damage the starch granules [42–44], thereby improving enzyme accessibility. Similar starch digestibility values have been reported when using semi-purified starch sources [45]. Despite high digestibility values, the intrinsic characteristics of WS and PS remained as reflected by a gradual decrease in *in vivo* starch digestibility as PS replaced WS in the diets. Statistical differences in starch digestibility were found for all areas of the small intestine, but the degree of difference decreased in more distal sections (from proximal jejunum to distal ileum the difference in the percentage of starch digestibility was: 9.1, 10.4, 3.7 and 2.3%, respectively). The separation between starch digestibility values was further reduced in the colon (0.7%) where statistical differences disappeared, suggesting that any starch remaining in the distal ileum had been fermented [46]. Therefore, starch digestibility values validated the dietary treatments used in this study to assess activation of L-cells by the amount of starch present in the digesta.

The digesta content pH in this trial was measured as a complementary indication of fermentation, under the assumption that lower pH values indicated higher SCFA concentration. Unlike SCFA findings which show an effect of PS level on SCFA concentration in all the three sections of the digestive tract measured, the results indicate that only crop pH was significantly affected by treatment, which followed a quadratic shape. Carbohydrate fermentation is associated with the production of SCFA, as well as CO_2_ and H^+^ [e.g. 47–49]. However, the digesta pH is the result of multiple factors including the fermentation of the mixture of nutrients present in the digestive tract [50], passage rate [51,52] and the composition of the digestive tract bacterial community [53]. Thus, it is possible that a combination of these factors contributed to a lack of correlation between some of the SCFA results and pH.

The concentration of PS directly affected total SCFA concentration in all three sections measured: crop, ileum and caeca. In the crop, it was found that the concentration of SCFA increased with PS level. Since the total amount of starch in diets was similar and one might predict equal or poorer fermentability for PS based on its digestive characteristics, the increase in total SCFA concentration could be an indication of longer retention time in the crop. The gizzard regulates the flow of digesta in the upper digestive tract [54]. When the ileal brake is activated, the release of PYY results in a reduction of gastric emptying and a decrease of passage rate [55,56] in mammals. Thus, an indication of longer retention time in the gizzard is supportive to ileal brake activation. In addition, although feed intake was not affected by PS level in this experiment [7], a linear increase in crop content with PS concentration was found, which also supports a decrease in passage rate as PS level increases, suggesting L-cells activation. The proportion of the major acids (acetic, propionic and lactic) in the crop was maintained, while minor acids (isobutyric and valeric) decreased. Branched SCFA are associated with protein fermentation [57], and consequently, these results suggest that as PS concentration increased, protein fermentation in the crop decreased.

The quadratic response found for total SCFA present in the distal ileum was unexpected as *in vivo* starch digestion showed a linear increase in the undigested starch content in this digestive tract section as dietary PS increased. Therefore, a linear increase in distal ileum SCFA was expected resulting from a direct relationship between the presence of starch and SCFA concentration. However, the quadratic response found in the current experiment is supported by unpublished broiler data from our lab (Savary et al., unpublished). Under similar experimental conditions, the total SCFA concentration found in the distal ileum digesta collected from 25 d old broilers fed graded levels of dietary PS (0, 25, 50, 75 or 100% PS) and challenged or not with 30x label dose of Coccivac-B52 showed a trend to change quadratically with PS level (*P* = 0.071), regardless of challenge. Although differences in lactic acid concentration were not significant due to the high degree of variability in the current trial, the effect of PS on the total amount of SCFA present in the distal ileum appears to be driven by the amount of lactic acid present (Table 6). It is possible that as PS increases, a shift in enteric microbiota towards lactic acid consuming species could result in a decrease of the amounts found for both lactic acid and total SCFA in the distal ileum. Alternatively, under similar fermentation levels, the higher digesta content found in the distal ileum could result in a decrease in concentration of SCFA for that section due to a dilution effect. Likewise, as both distal ileum and caeca empty weights increased with PS concentration, it is possible that the absorption rate of SCFA is increased, again resulting in a quadratic effect as SCFA concentration decreases. Interestingly, in the absence of dietary PS no lactic or butyric acid was found in the distal ileum, which supports digestibility data.

Total SCFA present in the caeca showed a quadratic response with PS, which appears to be driven by a sharp increase in the total amount of SCFA found in the caeca of birds fed 80 and 100% PS. Broilers in these two treatments showed an increase of 13 and 30% in the total amount of SCFA, when compared to 0% PS. Starch digestibility showed that most of the starch had been digested or fermented before reaching the caeca, except for the highest concentrations of PS, supporting the SCFA data. Changes in the SCFA composition in this section of the digestive tract suggest an effect of PS concentration on the type of bacteria present in the caeca. In fact, concentration of acids associated with protein fermentation (iso-butyric, iso-valeric and valeric acid; 50) decreased in 100% PS, while the contrary is true for others associated with starch fermentation (acetic and butyric acid; 50), which, similar to the results found in the crop, suggest a shift toward carbohydrate fermenting bacteria.

Evidence in mammals and chickens shows that SCFA are capable of activating L-cells [58–60]. For instance, Psichas and collaborators [60] found that intra-colonic administration of propionate stimulated the release of both GLP-1 and PYY in Wistar rats via the free fatty acid receptor 2. In chickens, L-cells are located in the jejunum and ileum [22,23], and the starch digestibility, pH and SCFA results from the current study indicate that L-cell activators are present in the distal small intestine of broiler chickens when dietary PS in included.

### Evaluation of L-cell activation

The proportional empty weight of every section of the digestive tract with the exception of the gizzard and duodenum increased linearly with PS concentration. Changes in crop empty weight could be associated with the increased amount of digesta found in this organ as dietary PS increased. It is possible that the increase in digesta content promoted crop muscle development in order to hold and evacuate digestive contents into the esophagus and proventriculus [54], explaining the increase in crop weight with PS. The digesta content weight in the jejunum and ileum also showed a linear increase with dietary PS, and thus the increase in empty weights of these sections could also be related to muscle development. However, these tissues were also proportionally longer. Similar results have been found for the pig's colon when animals are fed resistant starch [46,61]. Bird et al. [46] found a direct relationship between pig colon length and the amount of amylose present in the diet. These findings could be supportive of L-cell involvement, as in mammals GLP-1 and GLP-2 are co-secreted from L- cells [16]. In response to nutrient sensing, GLP-2 promotes intestinal growth [16,18,19], making it a potential player in the jejunum and ileum enlargement noted with increased dietary PS. An increase in empty weight and length of the caeca was also observed with increasing concentration of PS. Although no PYY or GLP-1 immunoreactive cells have been reported in this section, there is the possibility that these peptides could be affecting caecal length and weight through a neuroparacrine mechanism [62,63]. Alternatively, butyrate has been associated with intestinal health in broilers by promoting the growth of the digestive tract wall and decreasing inflammation [64]. As the amount of butyrate found in the caeca increased with PS concentration, it is possible that the observed changes in weight and length or the caeca are associated with this acid.

Neither feed intake [7], nor feeding behaviour [65] were affected by dietary PS in a trial conducted at the same time under identical conditions. However, crop content increased with dietary PS in the current trial, supporting a reduction in gastric emptying. Neuropeptides GLP-1 and PYY have both been shown to reduce gastric emptying in mammals when nutrients are sensed by L-cells [55,66]. The linear increase in crop content, as well as the higher SCFA concentration as PS increased in this organ’s contents, could be an indication of a lower rate of gastric (gizzard) emptying as a result of L-cell activation [54].

Like crop digestive content weight, jejunum and ileum relative digestive content also increased with PS concentration when controlling for differences in body weight and tissue length. Although the amount of soluble fibre was similar among diets, DF increased 3.4% from 0% PS to 100% PS in finisher diets due to a slightly lower starch content in the PS than was anticipated. The relative increase in fibrous material, along with the remaining amount of undigested starch and potential differences in water holding capacity could explain the change in small intestine content with increasing PS, without affecting feed intake [67]. Although the presence of a higher level of dietary PS could be partially responsible for the increase in small intestine content, the increase in total DF introduces a confounding factor that hinders interpretation of this particular result.

Average T50 obtained in this study was 490.5 min. Danicke et al. [68], using a similar methodology (adding TiO_2_ as an indigestible marker to the diets of 24 d old broilers), obtained an average T50 of 457 min, supporting our findings. An effect of PS level on average digesta passage time was found. However, this effect does not support a direct, linear relationship between PS level and ileal brake activation as the weight of digesta contents in the crop suggest. Contrary to our hypothesis, T50 decreased from 0 to 60% PS, and then increased in the two highest PS level dietary treatments. However, 80 and 100% PS T50 did not reach those obtained in diets containing 0 and 20% PS.

No effect of PS on the serum concentration of GLP-1 or PYY was found. It is possible that values measured as a result of collecting blood from the brachial (wing) vein are not sensitive to physiological changes because these peptides are released from the digestive tract enteroendocrine cells into the portal bloodstream. Collection of blood from the portal vein or from arterial blood may have been more appropriate. Furthermore, GLP-1 or PYY effects can be the result of both endocrine and paracrine action through the activation of the vagus nerve [69], so a lack of blood response does not rule out paracrine action. Nonetheless, the lack of a linear effect of PS level on passage rate suggests that secretion of GLP-1 or PYY did not differ among treatments.

Chicken serum GLP-1 concentrations obtained in this and other studies [70,71], are higher than normal concentrations reported in mammalian research [8–40 pmol/L; 9,72,73]. Kolodziejski and collaborators [70] reported GLP-1 serum levels in broilers of approximately 800 pg/mL (240 pmol/L), which are similar to those found in an experiment with laying hens using treatments and an experimental design similar to the current study (698 pg/mL; 71). Unlike the current study, a linear effect of dietary PS concentration was found on the laying hen serum concentration of GLP-1 [71]. Thus, it is possible that, at such high concentrations as found in the current study (1652 pg/mL or 496 pmol/L), all GLP-1 receptors are saturated, and so no effects on passage rate or feed intake are observed.

Similar to the ELISA results, gene expression analyses did not show an effect of PS on either proglucagon or PYY RNA expression levels, with the exception of a linear decrease in ileum proglucagon gene expression. Although it is important to remember that proglucagon is a gene that yields several peptides other than GLP-1, the lack of response in proglucagon B and the decrease in proglucagon in the jejunum with an increase in dietary PS does not support activation of the ileal brake by PS. It is possible that chickens respond in a different way to L-cell activation in comparison to the animals used in mammalian studies. A key difference between chickens and mammalian species is related to feeding behaviour. While the latter species eat discrete meals, broilers are fed *ad libitum* and feeding behaviour consists of short frequent meals [65,74]. Therefore, with the possible exception of the time at the end of an extended dark period (>8 h), broilers always have feed in the digestive tract [72,74,75]. Accordingly, it is possible that nutrient sensing mechanisms are continuously activated, resulting in the different physiological response. Serum GLP-1 concentrations reported in the current study appear to support this hypothesis.

The focus of the manuscript was to specifically assess the effect of starch digestion on ileal brake activation and data collected during this study reflects this decision. However, it must be recognized that other mechanisms can be involved in the production and physiological results of our work. For example, differences in the post-prandial glucose metabolism and/or synchronization with amino acid metabolism [4] might result in enhanced performance as reflected by the results obtained when broilers were fed a combination or starch sources that differ in the rate and extent of starch digestion [7].

## Conclusion

In conclusion, this study found evidence that the presence of starch and SCFA (L-cell activators) in the distal small intestine were affected by PS concentration. Dietary PS also promoted digestive tract tissue development and indirectly suggested reduced gastric emptying, indicative of L-cell activation. However, no direct evidence of ileal brake activation was found by measuring venous blood levels of GLP-1 or PYY, expression of proglucagon, proglucagon B and PYY genes in jejunal and ileal tissue or differences in T50.

## Supporting information

S1 Appendix (DOCX)

S1 Data (XLSX)
