# Supplementary material for: Assessing the effect of starch digestion characteristics on ileal brake activation in broiler chickens
Source: PLoS One. 2020 Feb 7;15(2):e0228647. doi: 10.1371/journal.pone.0228647 (PMC7006927; doi:10.1371/journal.pone.0228647)
Supplement: S1 Appendix — (DOCX) [file pone.0228647.s001.docx]

APPENDIX

| **Table A1**. Analyzed composition of dietary treatments (% DM) | | | | | | |
| --- | --- | --- | --- | --- | --- | --- |
|  | **0 PS** | **20 PS** | **40 PS** | **60 PS** | **80 PS** | **100 PS** |
| Starter | | | | | | |
| Dry matter | 90.99 | 91.48 | 91.18 | 91.22 | 91.14 | 91.20 |
| Total starch | 49.80 | 49.18 | 41.09 | 38.50 | 37.28 | 36.99 |
| Crude protein | 25.53 | 26.01 | 25.56 | 28.00 | 27.65 | 26.96 |
| Insoluble fiber | 13.42 | 14.82 | 18.20 | 19.04 | 22.83 | 20.42 |
| Soluble fibre | 2.91 | 2.53 | 2.87 | 2.62 | 2.94 | 2.78 |
| Fat | 4.67 | 4.50 | 4.70 | 4.66 | 4.53 | 4.36 |
| Ash | 6.32 | 6.47 | 6.44 | 7.07 | 6.89 | 7.07 |
|  |  |  |  |  |  |  |
| Grower |  |  |  |  |  |  |
| Dry matter | 90.72 | 91.43 | 91.71 | 91.00 | 90.52 | 91.00 |
| Total starch | 49.62 | 47.60 | 44.37 | 43.73 | 43.28 | 39.47 |
| Crude protein | 24.33 | 23.46 | 23.75 | 23.73 | 24.51 | 27.57 |
| Insoluble fiber | 12.93 | 10.26 | 12.70 | 13.38 | 13.59 | 17.15 |
| Soluble fibre | 3.18 | 2.28 | 2.79 | 2.76 | 2.29 | 2.34 |
| Fat | 5.24 | 4.89 | 5.02 | 4.86 | 4.93 | 4.90 |
| Ash | 5.89 | 5.68 | 5.82 | 5.91 | 6.16 | 6.41 |
|  |  |  |  |  |  |  |
| Finisher |  |  |  |  |  |  |
| Dry matter | 91.31 | 91.21 | 90.59 | 91.60 | 91.77 | 91.05 |
| Total starch | 47.61 | 47.36 | 46.63 | 45.34 | 42.43 | 41.68 |
| Crude protein | 23.03 | 24.56 | 24.53 | 24.88 | 25.68 | 23.55 |
| Insoluble fiber | 11.51 | 12.82 | 12.59 | 13.55 | 10.22 | 14.94 |
| Soluble fibre | 2.41 | 2.62 | 2.40 | 2.15 | 2.40 | 2.42 |
| Fat | 4.71 | 5.09 | 5.00 | 5.04 | 5.32 | 5.39 |
| Ash | 5.83 | 6.04 | 6.04 | 6.13 | 6.65 | 6.66 |
|  |  |  |  |  |  |  |
| All analyses were done in duplicate, with the exception of total starch, which was done in triplicate | | | | | | |
